# Identification of lactate regulation pattern on tumor immune infiltration, therapy response, and DNA methylation in diffuse large B-cell lymphoma

**DOI:** 10.3389/fimmu.2023.1230017

**Published:** 2023-09-18

**Authors:** Jinghua Wang, Yanjun Wang, Li Wan, Xinyuan Chen, Han Zhang, Shuo Yang, Liye Zhong

**Affiliations:** ^1^ Department of Hematology, Guangdong Provincial People’s Hospital (Guangdong Academy of Medical Sciences), Southern Medical University, Guangzhou, Guangdong, China; ^2^ Department of Urology, Sun Yat-sen University Cancer Center, State Key Laboratory of Oncology in South China, Collaborative Innovation Center for Cancer Medicine, Guangzhou, Guangdong, China; ^3^ Department of Endocrinology & Metabolism, Renmin Hospital of Wuhan University, Wuhan, Hubei, China; ^4^ Digestive Medicine Center, The Seventh Affiliated Hospital, Sun Yat-sen University, Shenzhen, Guangdong, China; ^5^ Department of Gastroenterology, Guangdong Provincial People’s Hospital (Guangdong Academy of Medical Sciences), Southern Medical University, Guangzhou, Guangdong, China; ^6^ Department of Gastroenterology, Peking University Shenzhen Hospital, Shenzhen, Guangdong, China

**Keywords:** lactate, tumor microenvironment, prognosis, DNA methylation, diffuse large B-cell lymphoma (DLBCL)

## Abstract

**Background:**

Lactate, produced through glycolytic metabolism in the tumor microenvironment (TME), is implicated in tumorigenesis and progression in diverse cancers. However, the impact of lactate on the remodeling of the TME in diffuse large B-cell lymphoma (DLBCL) and its implications for therapy options remain unclear.

**Method:**

A lactate-related (LAR) scoring model was constructed in DLBCL patients using bioinformatic methods. CIBERSORT, XCELL, and ssGSEA algorithms were used to determine the correlation between LAR score and immune cell infiltration. Tumor Immune Dysfunction and Exclusion (TIDE), rituximab, cyclophosphamide, adriamycin, vincristine, and prednisone (R-CHOP) cohorts, and Genomics of Drug Sensitivity in Cancer (GDSC) were utilized to predict the therapeutic response of DLBCL patients. The impact of the hub gene STAT4 on tumor biological behavior and DNA methylation was experimentally validated or accessed by the TSIDE database.

**Results:**

The LAR scoring model was developed based on 20 prognosis-related lactate genes, which enabled the division of DLBCL patients into high- and low-risk groups based on the median LAR score. Patients with high-risk DLBCL exhibited significantly worse survival outcomes in both the training cohorts (GSE181063) and the validation cohorts (GSE10846, GSE32918, and GSE69053), as indicated by statistically significant differences (all P<0.05) and area under the curve (AUC) values exceeding 0.6. Immune analyses revealed that low-risk DLBCL patients had higher levels of immune cell infiltration and antitumor immune activation compared to high-risk DLBCL patients. Furthermore, DLBCL patients with high LAR scores were associated with a lower TIDE value and poor therapeutic efficacy of the R-CHOP regimen. GDSC analysis identified 18 drugs that exhibited significant response sensitivity in low-risk DLBCL patients. Moreover, *in vitro* experiments demonstrated that overexpression of the lactate key gene STAT4 could suppress proliferation and migration, induce cell cycle arrest, and promote cell apoptosis in DLBCL cells. Transcriptional expression and methylation of the STAT4 gene were found to be associated with immunomodulators and chemokines.

**Conclusion:**

The lactate-based gene signature effectively predicts the prognosis and regulates TME in DLBCL. Our study underscores the role of lactate gene, STAT4, as an important tumor suppressor in DLBCL. Modulating STAT4 could be a promising strategy for DLBCL in clinical practice.

## Introduction

Diffuse large B-cell lymphoma (DLBCL) is a common lethal tumor, accounting for 40% of B-cell malignancies (1). Up to one-third of patients diagnosed with DLBCL experience relapse or incomplete remission following first-line treatment, with a low success rate observed in those undergoing salvage treatment regimens for relapsed DLBCL (2, 3). Notably, patients with identical histological type or cell of origin (COO) classification may exhibit markedly divergent clinical outcomes (4, 5), suggesting inadequacy of the current classification system in capturing the biological heterogeneity of DLBCL. Therefore, the clinical development of predictive gene expression profiling (GEP) markers that can complement COO is urgently needed.

Lactate is produced primarily in aerobic glycolysis, and many are released into the extracellular space, thus entering the tumor microenvironment (6). Several studies showed that the acidic environment formed by a high lactate concentration account for tumor cell metastasis, angiogenesis, and treatment resistance (7, 8). For example, activation of lactate-related gene HK2, a key metabolic driver of DLBCL (9), significantly increases cellular lactate production and promotes tumor cell invasion, metastasis, and drug resistance (10). IL18 (11), another molecule significantly associated with lactate levels, was significantly associated with immunosuppression and poor prognosis in DLBCL patients (12). However, these lactate-related genes have not been successfully integrated into the prognostic scoring systems to better reveal the impact of lactate in the tumor microenvironment on the pathogenesis and clinical outcome of DLBCL.

**Scheme 1:**
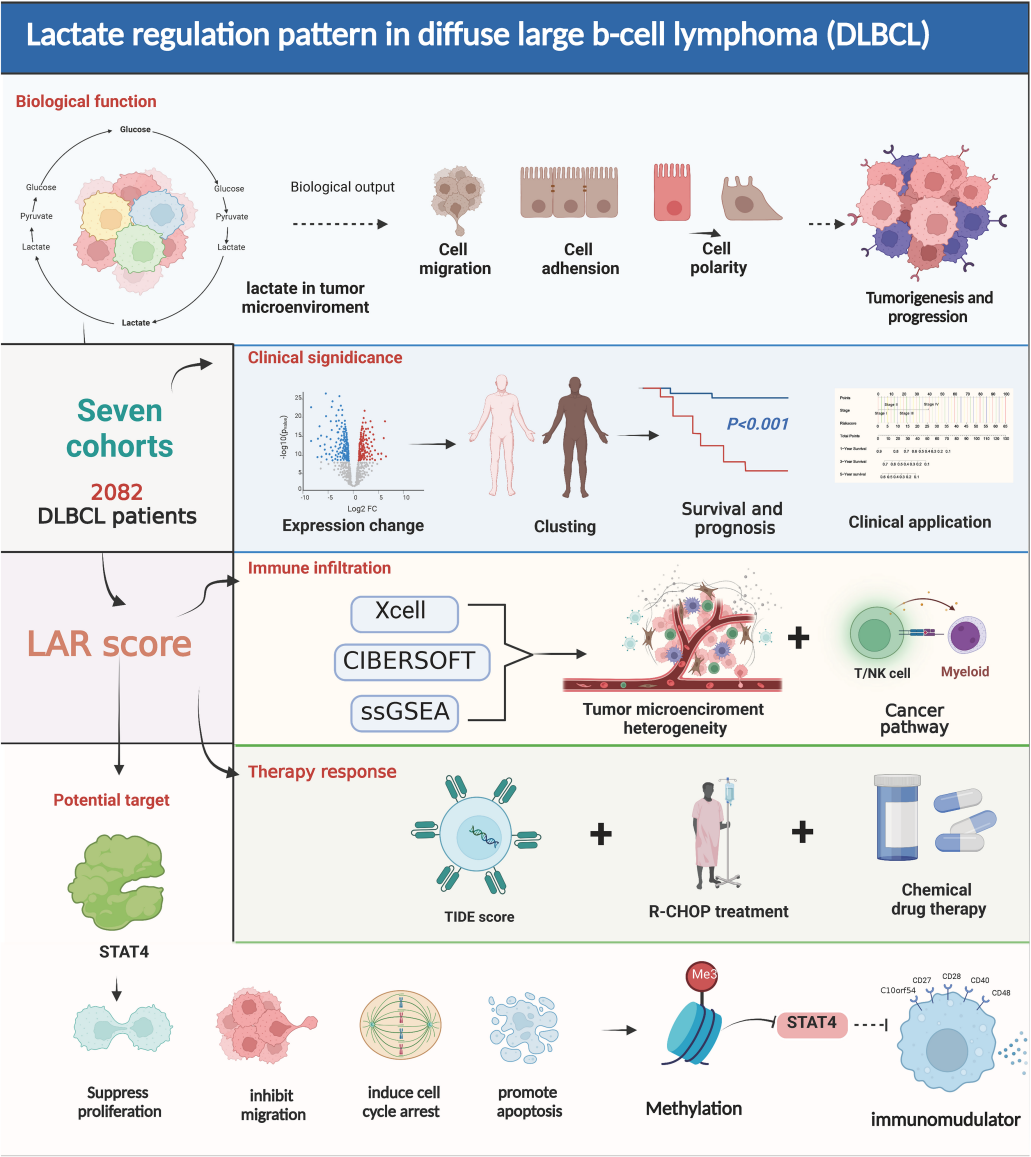
Lactate regulation pattern in diffuse large B-cell lymphoma.

In this study, we first proposed a lactate-related score (LARscore) prognostic scheme in DLBCL patients, which was used to predict prognosis, molecular subtype distribution, immune infiltration level, rituximab, cyclophosphamide, adriamycin, vincristine, and prednisone (R-CHOP) response, and sensitivity to some chemotherapy drugs. Then, we investigated the effects of lactate key gene STAT4 on the malignant biological behavior of DLBCL cells. Finally, the relationship between key gene STAT4 transcript expression, DNA methylation, and immunomodulators was assessed by the TISDB online database. The graphical abstract was shown in **Scheme 1**. The comprehensive characterization of these lactate genes within the tumor microenvironment provides insights into tumor immune infiltration and may have significant implications for improving personalized cancer treatment by targeting lactate genes.

## Methods

### Data collection

The transcriptome datasets GSE181063, GSE10846, GSE32918, GSE69053, GSE32018, and GSE56315, were downloaded from the Gene Expression Omnibus (GEO) database using the “getGE” function of R package “GEOquer”. Among them, GSE32018 and GSE56315 cohorts were used for control analysis between tumor and normal samples, and the R package”sv” function combat was used to remove the batch effect. GSE181063 was used as the training set to construct the LARscore system, and GSE10846, GSE32918, and GSE69053 datasets were used as the validation sets. The DLBCL transcriptome data, mutation data, copy number variation, and clinical information data were collected from the The Cancer Genome Atlas (TCGA)-DLBCL dataset for Single Nucleotide Variation (SNV) and Copy Number Variation (CNV) correlation analysis. At the same time, we analyzed the drug response based on GDSC database. We collected lactate-related genes from the literature in **Table S1** and interleaved the above gene sets to obtain a gene set containing 208 genes. We defined this gene set as the lactate-related gene set.

### Identification and enrichment analyses of differentially expressed genes

The R package “limma” was utilized to detect differentially expressed genes (DEGs) between DLBCL and its reference samples, with a screening threshold established as adj-P value< 0.01, |log2 Fold Change| > 1.5. Subsequently, the DEGs were subjected to Gene Ontology (GO) and Kyoto Encyclopedia of Genes and Genomes (KEGG) enrichment analysis through the “clusterProfile” package of R, with statistical significance determined at P< 0.05.

### Mutation and copy number variant analysis

To detect the variation of lactate gene in DLBCL, we performed a SNV analysis using the “maftools” package of R and CNV analysis using “Gistic2” package of R. Survival analysis was performed using the “survival” package of R, and Kaplan-Meier (KM) curves were plotted by the “ggsurvplot” package of R to access the survival difference.

### Establishment of the prognostic model

To construct the prognostic model, Cox regression analysis was conducted on the lactate genes of the DLBCL samples using the ‘coxph’ function of the ‘survival’ package. Univariate regression analysis was used to identify genes that significantly affect the prognosis, while multivariate Cox analysis was used to determine the genes independently related to the prognosis. Least Absolute Shrinkage and Selection Operator (LASSO) regression analysis was performed using the “cv. gimne” function of “lar” package for genes with *P*<0.01 in two Cox analyses. The LASSO regression algorithm was used to screen genes whose coefficients were unequal to 0, and an optimal prognostic scoring model for lactate was constructed. LARscore was defined by summing the product of lasso coefficient and gene expression.


$$\text{LARscore}=\sum_{i=1}^{n}expression\;of\;gene\;i\;*lasoo\;coefficient\;of\;gene\;i$$

The DLBCL groups were classified based on the median risk score. A high LARscore group was defined as having a LARscore higher than this value, while a low LARscore group was characterized by a LARscore lower than this value. The R package “timeROC” was used for ROC analysis, and the “survival” package was used to visualize the results of prognostic genes and clinical feature factors.

### Decision curve analysis (DCA)

DCA is a statistical method used to assess the clinical utility of diagnostic or predictive models. It helps in determining the net benefit of using a particular model across different thresholds of decision-making. In DCA, the “None” and “ALL” reference lines are used as benchmarks for comparison. Under the same probability, the clinical usefulness was better when the net benefit was higher.

### Analysis of immune infiltration

Cibersort, ssGSEA, and xCell algorithms were used to quantify the proportion of immune cells in the TME. **Table S2** presents the gene sets used by ssGSEA. The Tumor Immune Dysfunction and Exclusion (TIDE) score was computed utilizing the TIDE software to forecast the clinical efficacy of immune checkpoint inhibitor (ICI) therapy. The immune score was calculated using the R package “estimate”.

### Response to R-CHOP treatment and drug sensitivity analysis

We selected two cohorts treated with R-CHOP (GSE181063 and GSE10846) to predict the response to R-CHOP treatment. Due to the lack of information on efficacy, we used the overall status to evaluate efficacy. Patients who received treatment and survived were considered to be in the R-CHOP-response group, and patients who died were supposed to be in the R-CHOP-nonresponse group. To gain a deeper comprehension of the influence of LARscore on drug response, an investigation was conducted on the correlation between LARscore and drug response of tumor cell lines in the Genomics of Drug Sensitivity in Cancer (GDSC) database, utilizing Spearman correlation analysis. Furthermore, an analysis was performed on the signaling pathways of genes targeted by these drugs.

### Cell culture

The normal B cell line GM12878 and three DLBCL cell lines (SU-DHL-2, OCI-LY19, DB) were cultured in high glucose RPMI-1640 (Gibco, USA) containing 10% fetal bovine serum (Gibco, USA). Incubator conditions were set at 37°C and 5% CO_2_.

### Total RNA extraction and quantitative reverse transcription polymerase chain reaction

In accordance with the Declaration of Helsinki, peripheral blood samples were obtained from patients with newly diagnosed DLBCL at Guangdong Provincial People’s Hospital, with informed patient consent and medical information collection. The Ethics Committee of the hospital approved this study. Total RNA was extracted using the E. Z. N. A. Total RNA Isolation Kit (Omega, GA, United States). The PrimeScriptTM RT-PCR kit (TaKaRa, Otsu, Japan) was utilized for the generation of cDNAs from reverse transcription. The qRT-PCR was conducted using SYBR Premix Ex Taq (TaKaRa, Otsu, Japan) in accordance with the manufacturer instructions of Biorad CFX Connect (Bio-Rad Laboratories, CA, United States). ABL was used as an internal control gene. The primers of IL-18, GPI, and STAT4 are as follows: IL-18 forward (5’-CAAGGAAATCGGCCTCTATTTG-3’), reverse (5’-CCTCTAGGCTGGCTATCTTTATACATACT-3’); GPI forward (5’-GCGCCGCCTCTTCGAT-3’), reverse (5’-TGCCCATGGTTGGTGTTG-3’); STAT4 forward (5’-GCAGCAAATCGCCTGCAT-3’), reverse (5’-CTGCCAATAGTGTAAAGCAGTTCTG-3’).

### Protein extraction and western blot

The cell lines’ proteins were lysed using RIPA cell lysis buffer supplemented with protease inhibitors and phosphatase inhibitors, and the quantification of total protein was performed using the BCA method. Subsequently, the protein samples were subjected to electrophoresis on 10% SDS-PAGE gels, transferred onto PVDF membranes, and blocked with 5% skim milk. The membranes were then incubated with primary antibodies overnight at 4°C, followed by three washes and incubation with the corresponding secondary antibody for 1 hour. Finally, the membranes were exposed using the instrument after the addition of a chemical chromogenic solution.

### Cell transfection

Construct the overexpression vector, and two cell lines (SU-DHL-2 and OCI-LY19) with differential expression of the target gene were selected for transient transfection, and the transfection efficiency was detected by qRT-PCR and western blot.

### Flow cytometry analysis

After staining with the AnnexinV-FITC/PI apoptosis detection kit and the cell cycle staining kit following the manufacturer’s instructions, the cells were analyzed for apoptosis and cell cycle distribution using flow cytometry. Specifically, AnnexinV-FITC and PI staining were used to distinguish between early and late apoptotic cells, and the cell cycle staining kit enabled the identification of cells in G0/G1, S, and G2/M phases.

### Detection of lactate levels

To measure lactate levels, the lactate concentration in the supernatant of lymphoma cells, empty vector lymphoma cells, and lymphoma cells overexpressing the STAT4 gene was determined using a lactate assay kit. The experiment was performed in triplicate under the same cell density conditions.

### DNA methylation

Relations between immunomodulators, MYC molecules, chemokines (or receptors) and expression, and DNA methylation of STAT4 were assessed by the TISDB online database (http://cis.hku.hk/TISIDB). In this tab, users can examine which immunomodulators and chemokines (or receptors) might be regulated by STAT4 and STAT4 methylation.

### Statistical test

We used R software (version 4.1.0) and SPSS software (version 25.0) for statistical analysis. Difference analyses were performed using the Wilcoxon test. Significance levels varied across analyses. Correlation analysis was performed using Spearman correlation. The correlation test was performed using the R language cor. test function. All *in vitro* experiments were repeated three times. P<0.05 in correlation statistics was considered significant.

## Results

### Biological characteristics of lactate-related genes in DLBCL

**Figure 1:**
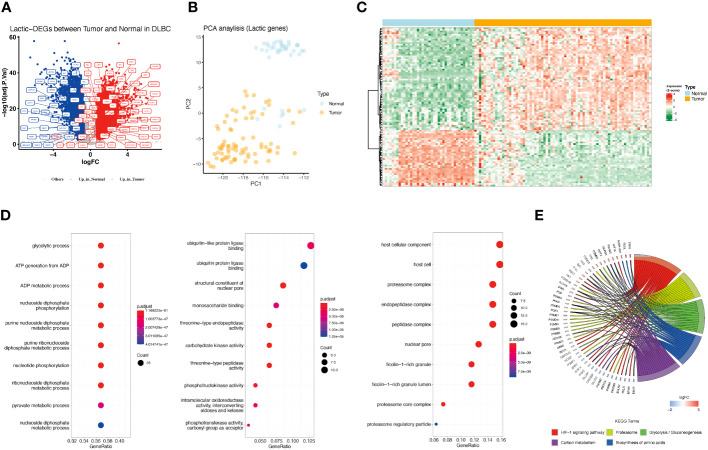
Differences in lactate-related gene expression in normal and tumor samples and its enrichment analysis. **(A)** Differentially expressed lactate-related genes between normal and DLBCL. Red represents genes highly expressed in tumors, and blue represents genes highly expressed in normal tissues. Gray represents no difference in expression. Lactate-related genes are annotated as tags. **(B)** PCA analysis showed that the expression of lactate-related genes represents distinct lactate phenotypes. Yellow represents tumor samples, and blue represents normal samples. **(C)** Gene expression profile of lactate-related DEGs between normal and tumor samples. **(D)** Bar charts showing enriched GO function, including Biological Process (BP), Molecular Function (MF), and Cellular Component (CC). **(E)** Chordal graph showing enriched KEGG pathway.

There were 208 lactate-related genes identified in GSE32018 and GSE56315, but 96 genes showed significant differences in expression between DLBCL and normal tissues (**Figures 1A, C**; **Table S3**). Among them, 34 genes were highly expressed in normal samples, and 62 genes were highly expressed in DLBCL samples. Principal Component Analysis (PCA) based on 208 lactate genes found that lactate-related genes could well distinguish tumor samples from normal samples (**Figure 1B**). Consequently, functional enrichment analysis of 96 lactate-related DEGs showed that the lactate-related subnetwork mainly involved in the glycolytic process, HIF-1 signaling pathway, which are closely related to the production of lactate and the tumor development (**Figures 1D, E**).

### Genomic variations of lactate-related genes and their impact on prognosis

**Figure 2:**
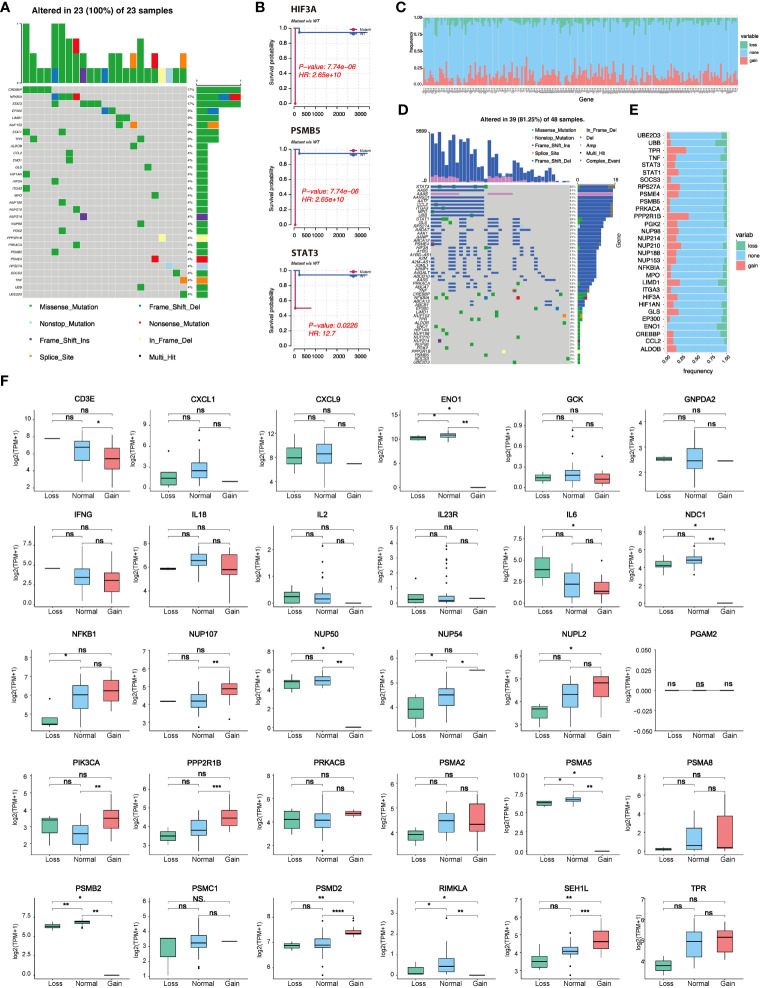
Lactate-related gene mutation, CNV, and prognostic analysis. **(A)** Waterfall plot showing mutations of lactate-related genes. **(B)** Three lactate genes exhibited significant differences in prognosis between the mutant and wild-type groups. **(C)** CNV statistics of all lactate-related genes. **(D)** Presentation of top 50 genes with CNV by waterfall plot. **(E)** Statistics of types of CNV in lactate-related genes with mutations. **(F)** Correlation analysis between the expression of lactate-related genes and different CNV subtypes. *P<0.05, **P<0.01, ***P<0.001, ****P<0.0001. ns, not significant.

Based on the TCGA-DLBCL dataset, we performed the correlation analysis of SNV and CNV. Mutations of lactate-related genes were detected in 23 samples. 30 lactate-related genes were mutated (**Figure 2A**). Among the mutated lactate-related genes, significant differences in prognosis were observed between the mutant and wild-type genes in three genes including HIF3A, PSMB5, and STAT3 (**Figure 2B**). Furthermore, we analyzed the prognostic difference between lactate-related gene mutation samples and wild-type samples in all samples. The results indicated that while the samples with lactate-related gene mutations had a relatively poorer prognosis than the wild-type samples, the difference was found to be statistically insignificant (**Figure S1**). Second, we used GISTIC2 method to analyze the CNV of TCGA-DLBCL samples. According to statistics, CNV events occurred in 196 lactate genes among 208 lactate-related genes, and the gain proportion was greater than the loss (**Figure 2C**). **Figure 2D** shows the top 50 lactate genes with CNV events. Among them, 30 mutated genes also had CNV, and the gain proportion was greater than the loss in mutated genes (**Figure 2E**). Furthermore, among the 30 lactate-related mutated genes, except PGAM2, the CNV of most genes showed a positive correlation with the expression level (**Figure 2F**), suggesting that genetic variation is an essential factor affecting the expression of lactate molecules.

### Identification of lactate-related gene prognostic model

**Figure 3:**
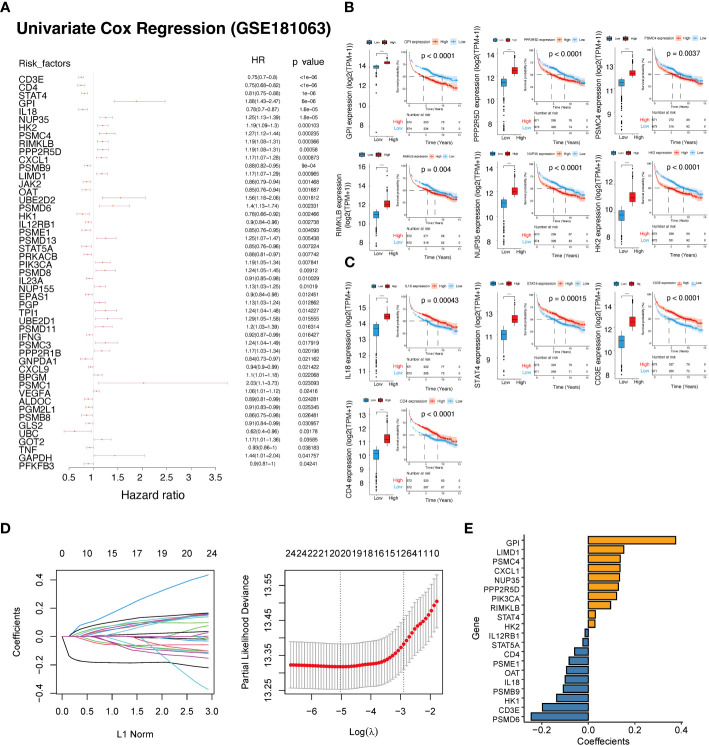
Construction of a prognostic scoring system based on lactate-related genes. **(A)** Forest plot showing the results of univariate Cox regression analysis of lactate-related genes. **(B)** KM curves and expression of six genes with HR >1. **(C)** KM curves and expression of four genes with HR<1. **(D)** The coefficients and lambda parameters obtained through lasso regression analysis was presented using the LASSO Cox regression model. **(E)** The coefficients of the lasso significant genes used in the calculation of the lactate-related prognostic score. ****P<0.0001.


By employing univariate Cox regression analysis on the GSE181063 dataset, we investigated the association between 208 lactate-related genes and the overall survival. Our findings revealed that 49 genes showed a significant correlation with the survival, where 25 genes had a P-value<0.01 in the univariate Cox regression analysis (**Figure 3A**; **Table S4**). Subsequently, we plotted the survival curves for six genes that had HR>1 (HR rank top 6) between the high and low expression groups (**Figure 3B**), and four genes with HR<1 (HR rank bottom 4), respectively (**Figure 3C**). Furthermore, we developed a lactate-related gene prognostic model using the GSE181063 dataset as a training set. After performing multivariate regression analysis on the 49 genes, we observed that all 49 genes had a P-value<0.05, out of which 26 genes had a P-value<0.01. To minimize the number of genes for downstream analysis, we employed a criterion of P<0.01, and obtained the intersection of 24 significant genes identified in both univariate and multivariate Cox regression analyses. The 24 prognostic genes were further narrowed down using the LASSO algorithm, which reported 20 risk genes (**Figure 3D**), including CD3E, CD4, STAT4, GPI, IL18, NUP35, HK2, PSMC4, RIMKLB, PPP2R5D, CXCL1, PSMB9, LIMD1, OAT, PSMD6, HK1, IL12RB1, PSME1, STAT5A, PRKACB, and PIK3CA (**Table S5**). Based on the lasso coefficients (**Figure 3E**), we calculated the LAR score and constructed a novel prognostic scoring model. The LAR score was presented as follows:

$$\begin{array}{c} \text{LAR score }=\;\left(0.189761897\times \text{CD}3\text{E}\right)\;+\;\left(0.055267499\times \text{CD}4\right)\;+\\ \;\left(0.029631704\times \text{STAT}4\right)\;+\;\left(0.366223851\times \text{GPI}\right)\;+\;\\ \left(0.096863197\times \text{IL}18\right)\;+\;\left(0.139596747\times \text{NUP}3\;5\right)\;+\;\\ \left(0.027248505\times \text{HK}2\right)\;+(0.129855681\times \text{PSMC}4)\;+\\ \left(0.098117054\times \text{RIMKLB}\right)\;+\;\left(0.130784925\times \text{PPP}2\text{R}5\text{D}\right)\\ \;+\;(0.131483148\times \text{CXCL}1)\;+\;\left(0.106748892\times \text{PSMB}9\right)\\ \;+\;(0.149933349\times \text{LIMD}1)+\;\left(0.084742819\times \text{OAT}\right)\\ \;+\;\left(0.230937617\times \text{PSMD}6\right)\;+\left(0.138908288\times \text{HK}1\right)\;+\\ \;\left(0.013079607\times \text{IL}12\text{RB}1\right)\;+\;\left(0.07630056\times \text{PSME}1\right)\\ \;+\;\left(0.024127912\times \text{STAT}5\text{A}\right)\;+\;\left(0.037228828\times \text{PRKACB}\right)\\ \;+\;\left(0.130347012\times \text{PIK}3\text{CA}\right) \end{array}$$

### The lactate-related risk score model exhibits a high predictive capacity and robustness in stratifying cancer prognosis

**Figure 4:**
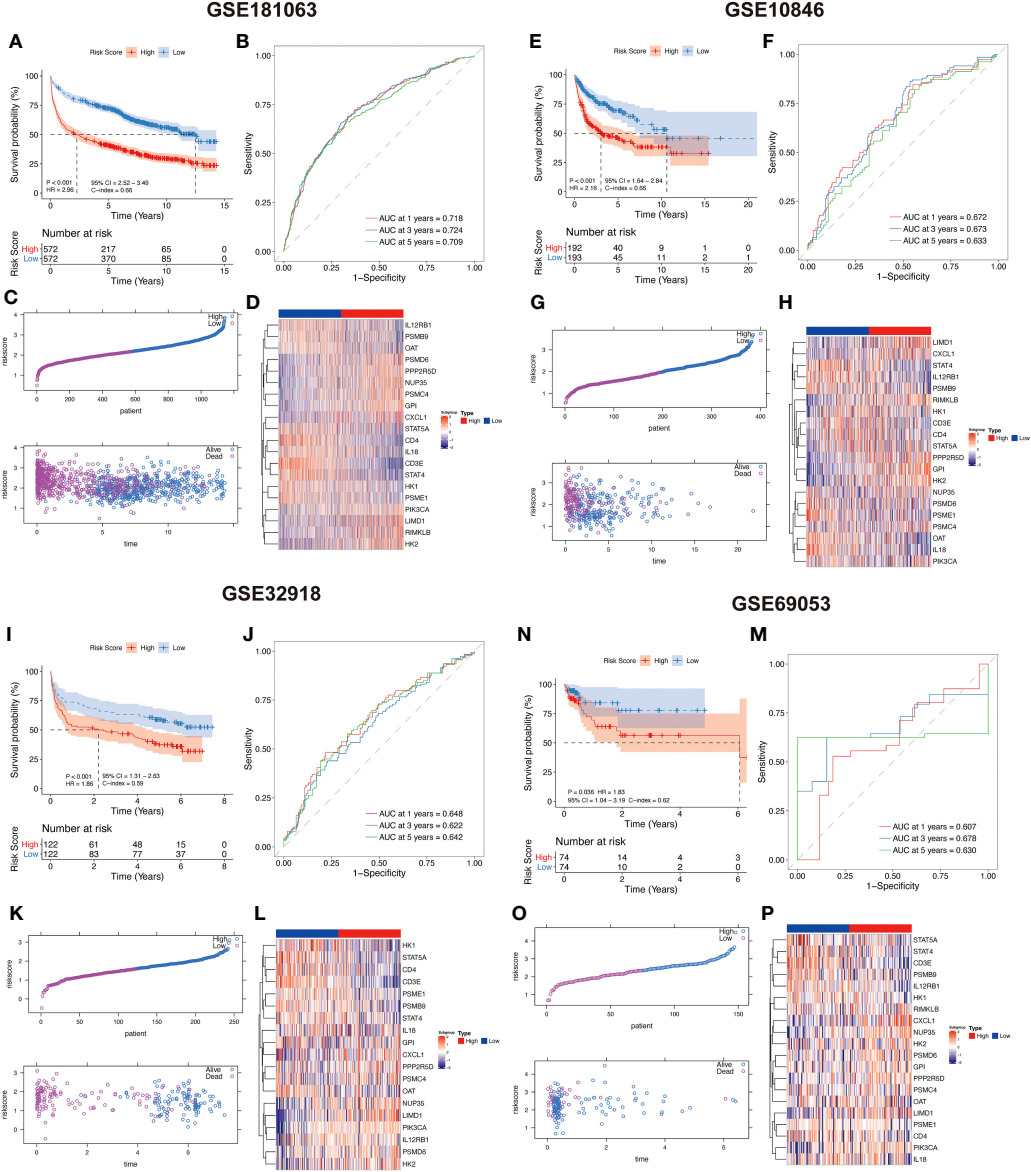
Lactate-related prognostic scoring system showing a high predictive performance in DLBCL prognosis. **(A, E, I, N)** KM curves showing that high LAR scoring group had a worse survival compared with low LAR group in GSE181063, GSE10846, GSE32918, and GSE69053 datasets. **(B, F, J, M)** ROC curves of 1, 3, and 5 years in GSE181063, GSE10846, GSE32918, and GSE69053 datasets. **(C, G, K, O)** Distribution of LAR score and its correlation with sample survival status were evaluated in GSE181063, GSE10846, GSE32918, and GSE69053 datasets. **(D, H, L, P)** Expression profile of 20 risk genes between high and low-LAR score groups in GSE181063, GSE10846, GSE32918, and GSE69053 datasets.

Based on the median LAR score, DLBCL samples were categorized into two groups, namely the high LAR score group and the low LAR score group. In the GSE181063 dataset, patients belonging to the low LAR score group exhibited significantly better survival outcomes compared to those in the high LAR score group (P<0.001) (**Figure 4A**). The ROC curve analysis revealed that the AUC values for 1, 3, and 5 years were 0.718, 0.724, and 0.709, respectively (**Figure 4B**). With the increase in LAR score, the survival time of the patients decreased, and the number of deaths increased significantly (**Figure 4C**). The expression of 20 LAR genes was also significantly different between the high- and low-LAR score group (**Figure 4D**). To assess the robustness of the lactate-related scoring model in cancer prognosis stratification, we compared the prognostic differences between the LAR score groups using multiple external datasets, including GSE10846, GSE32918, and GSE69053. We found that the LAR model had significant prognostic stratification efficiency on all three datasets. Specifically, both the high and low LAR score groups showed significant prognostic differences in the three datasets (all *P*<0.001) (**Figures 4E, I, N**). The AUC values of three datasets at 1, 3, and 5 years were all higher than 0.6 (**Figures 4F, J, M**). The survival time of patients significantly decreased and the number of deaths increased with the increase in risk score in all three datasets (**Figures 4G, K, O**). The expression levels of the 20 genes included in the model were found to differ significantly between the LAR risk high and LAR risk low groups across all three datasets (**Figures 4H, L, P**).

Moreover, we employed DCA curve to assess the comparative performance of our risk prediction model in relation to published model (as shown in **Figure S2**). Upon comparing our model with four other previously published models (13–16), we evaluated the total survival rates over distinct time horizons—1 year, 2 years, 3 years, and 5 years. Notably, our risk model consistently exhibited higher vertical axis values across a wide spectrum of decision thresholds, suggesting its potential to yield improved clinical decision-making outcomes in DLBCL.

### Clinical significance of the LAR score

**Figure 5:**
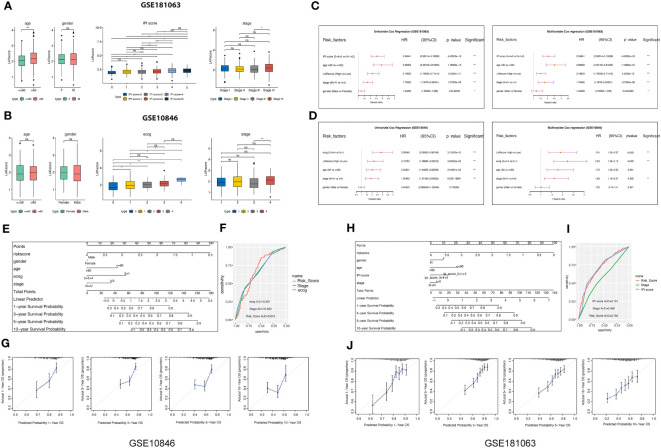
Correlation analysis between LARscore and clinical features. **(A)** Differences in the LAR score between various clinicopathological features in the GSE181063 dataset. IPI: International Prognostic Index. **(B)** Differences in the LAR score between various clinicopathological features in the GSE10846 dataset. **(C)** Univariate and multivariate Cox analysis of LAR score and clinical features in the GSE181063 cohort. **(D)** Univariate and multivariate Cox analysis of LAR score and clinical features in the GSE10846 cohort. **(E)** The nomogram developed based on the GSE10846 dataset. **(F)** ROC curve of the clinical factors in the GSE10846 dataset. **(G)** Calibration of the model from 1 to 10 years in the GSE10846 dataset. **(H)** The nomogram developed based on the GSE181063 dataset. **(I)** ROC curve of the clinical factors in the GSE181063 dataset. **(J)** Calibration of the 1–10 years model in the GSE181063 dataset. *P<0.05, **P<0.01, ***P<0.001, ****P<0.0001. ns, not significant.

To examine the clinical significance of the LAR score, we conducted a comparative analysis of the variations in LAR score, categorized by distinct clinical characteristics, in GSE181063 and GSE10846 datasets. Our findings indicate that patients with advanced stage, high-risk International Prognostic Index Score (categorizing DLBCL patients as low, intermediate, or high-risk), and higher ECOG score (ranging from 0 to 5 indicating varying levels of functional impairment) exhibit a higher LAR score (**Figures 5A, B**). In both datasets, the LAR score has significant prognostic predictive efficiency and is an independent prognostic factor in both univariate and multivariate Cox analyses (**Figures 5C, D**; **Tables S6-S7**). Based on the LAR score and multiple clinical characteristics, we constructed a nomogram for the GSE10846 cohort (**Figure 5E**) and GSE181063 (**Figure 5H**) to guide clinical practice. In the two nomograms, the AUC values of LAR score were higher than those of grade and stage, indicating that LAR score had better prediction efficiency than the traditional Ann Arbor staging (**Figures 5F, I**). **Figures 5G–J** present the calibration curves of LAR score model at 1, 3, 5, and 10 years, and the result indicate that the nomogram is a worthwhile mode for predicting DLBCL patient prognosis in both the short and long term.

### Immune mechanisms and pathway correlations in the lactate prognostic scoring system of DLBCL

**Figure 6:**
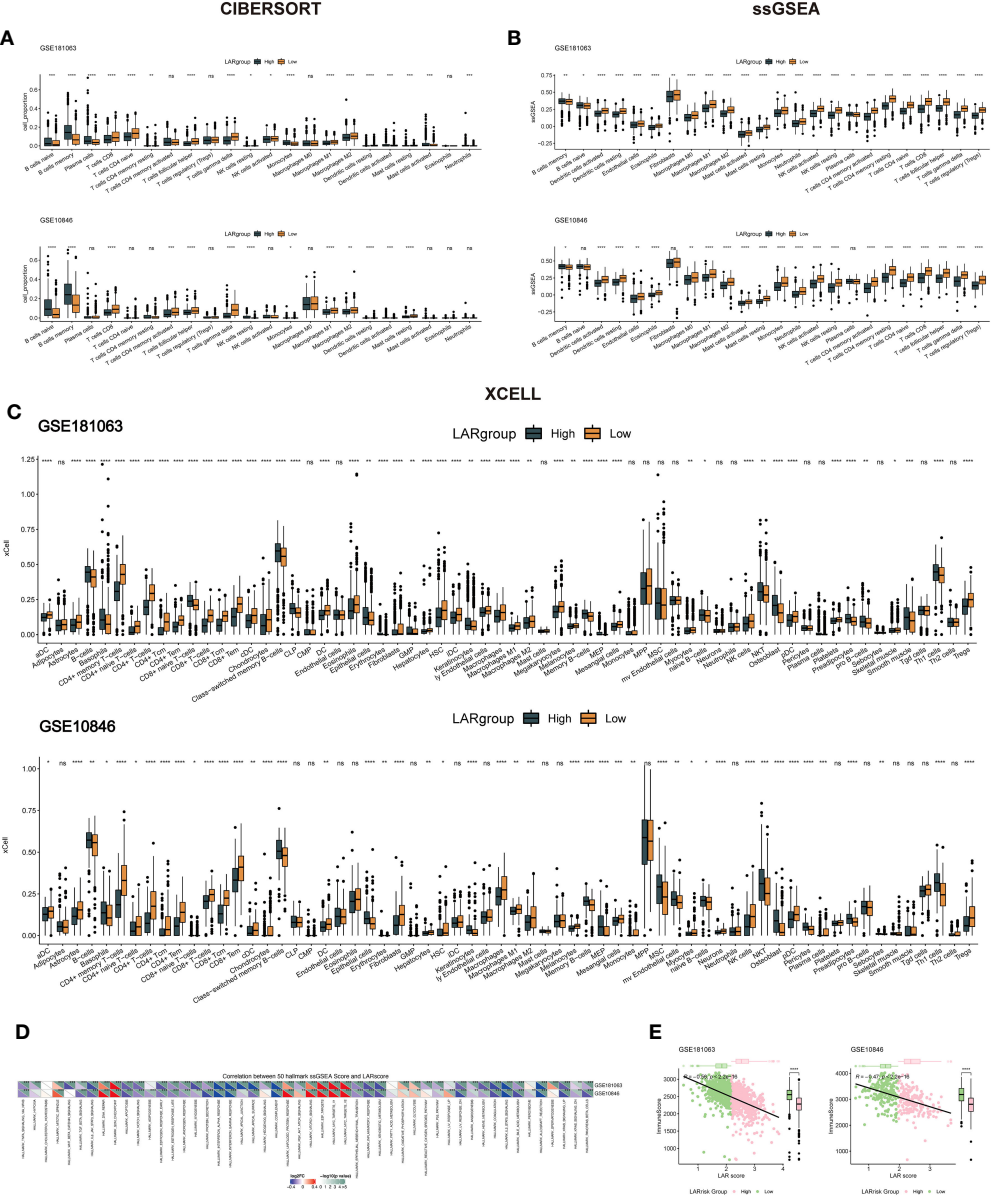
Immune infiltration levels and cancer-related pathway analysis in DLBCL. **(A)** Immune cell infiltration analysis using CIBERSORT for the GSE181063 and GSE10846 cohorts. **(B)** Immune cell infiltration analysis using ssGSEA for the GSE181063 and GSE10846 cohorts. **(C)** Immune cell infiltration analysis using Xcell for the GSE181063 and GSE10846 cohorts. **(D)** Enrichment analysis of the 50 cancer hallmark pathways using ssGSEA in the GSE181063 and GSE10846 cohorts. **(E)** Correlation analysis between LAR score and immune score in the GSE181063 and GSE10846 datasets. *P<0.05, **P<0.01, ***P<0.001, ****P<0.0001. ns, not significant.

In order to understand the potential mechanisms affecting the lactate prognostic scoring system in DLBCL, immune cell infiltration in the GSE181063 and GSE10846 cohorts was analyzed using CIBERSORT (**Figure 6A**), ssGSEA (**Figure 6B**) and XCELL (**Figure 6C**) algorithms. The differences in most immune cells between the two groups were significant, suggesting that the lactate score was closely related to tumor immune infiltration. In the three different algorithms, we can see that the cells associated with antitumor immunity, including CD8+T cells, activated dendritic cells, naive CD4+T cells, and activated NK cells, were significantly enriched in the low LAR score group. Next, based on the 50 cancer hallmark pathways, we calculated the enrichment scores of ssGSEA for the cohorts GSE181063 and GSE10846 and performed the spearman correlation test. Our results showed that the LAR score had a significant positive correlation with the enrichment scores of multiple pathways, such as MYC and MTORC (**Figure 6D**), suggesting that multiple molecular mechanisms affect the lactate scoring system. Finally, we calculated the sample immune score using an estimate tool and evaluated the relationship between the LAR score and the immune score using spearman correlation. We found a significant negative correlation between the LAR score and immunity score in both datasets (**Figure 6E**). The immune score for the high LAR score was significantly lower than that for the low LAR score, which further suggested that the immune level of DLBCL decreased significantly with an increased LAR score.

### Lactate risk score predicts R-CHOP treatment efficacy and chemosensitivity

**Figure 7:**
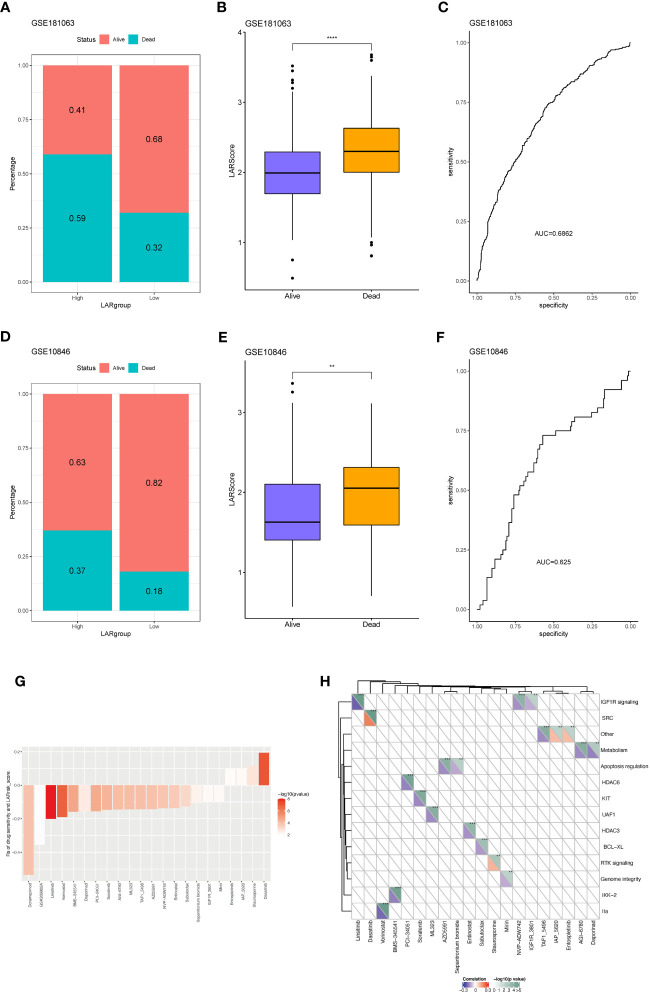
Predicting R-CHOP treatment efficacy and chemotherapy sensitivity using the lactate prognostic score system. **(A)** Distribution of samples with differential drug response between high and low-risk groups in the GSE181063 dataset. **(B)** Variation in LAR scores among samples with differential drug response in the GSE181063 dataset. **(C)** Area under the receiver operating characteristic (AUROC) curve for drug response. **(D)** Distribution of samples with differential drug response between high and low-risk groups in the GSE10846 dataset. **(E)** Variation in LAR scores among samples with differential drug response in the GSE10846 dataset. **(F)** AUROC curve for drug response. **(G)** Significant correlation between drug IC50 and LAR score. **(H)** Drug-associated pathways.

To investigate whether there is a difference in the TIDE values between groups at high- and low-risk, four datasets (GSE181063, GSE10846, GSE32918, and GSE69053) were analyzed by the TIDE algorithm. As shown in **Figures S3A–D**, no significant differences were observed in TIDE scores between the high- and low-risk groups across the four cohorts. However, a negative correlation was found between the LAR score and TIDE in three cohorts (excluding GSE32918) (**Figure S3E**), underscoring the intricate interplay between molecular characteristics, immune responses, and treatment outcomes in these contexts. Two cohorts that received R-CHOP treatment (GSE181063 and GSE10846) were selected to assess the therapeutic efficacy between the high- and low-LAR score groups. The results demonstrated that patients with a low LAR score exhibited a better therapeutic response in both cohorts (**Figures 7A, D**). Notably, significant differences in LAR scores were observed between the R-CHOP-effective and R-CHOP-ineffective groups, with higher LAR scores observed in the latter group (**Figures 7B, E**). The AUC of ROC curves for LAR score and efficacy were all greater than 0.6 (**Figures 7C, F**). Furthermore, the association between the LAR score and chemotherapeutic drug response was explored by analyzing the correlation between the LAR score and drug sensitivity in tumor cell lines using the GDSC database. The results identified 22 significant correlations between the LAR score and drug sensitivity. Among them, four drug pairs showed a positive correlation with the LAR score (Entospletinib, IAO 5620, Staurosporine, and Dasatinib), while 18 drug pairs showed an inverse correlation with the LAR score (Doramapimod, GSK269962A, Linsitinib, Vorinostat, BMS-345541, Daporinad, PCl-34051, Sorafenib, AGl-6780, ML323, TAF1 5496, AZD5991, NVP-ADW742, Entinostat, Sabutoclax, Sepantronium bromide, lGF1R, and 3801Mirin) (**Figure 7G**). Moreover, the signaling pathways targeted by these drugs were analyzed, revealing that drugs associated with high LAR scores mainly targeted the SRC and RTK signaling pathways, while drugs associated with low LAR scores mainly targeted IGF1R, apoptosis, and lla signaling pathways (**Figure 7H**). These findings collectively suggest that the LAR score is a potential biomarker for determining optimal therapeutic strategies by correlating with drug sensitivity.

### The overexpression of the lactate key gene STAT4 inhibited the proliferation and migration of DLBCL cells

**Figure 8:**
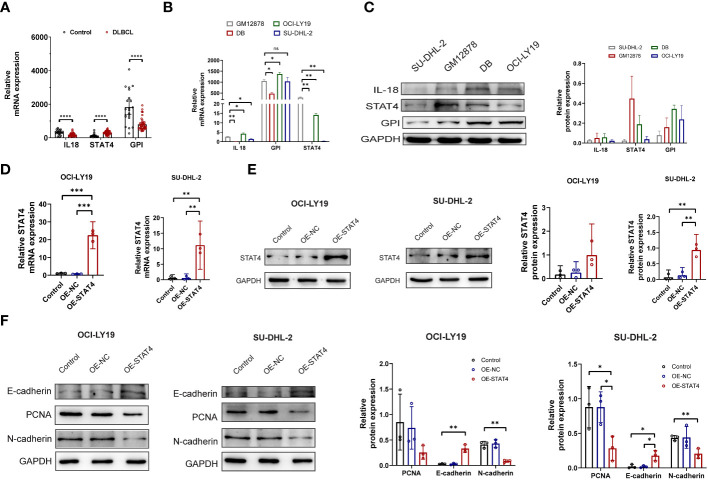
Overexpression of STAT4 suppresses DLBCL cell proliferation, migration, and promotes apoptosis. **(A)** mRNA expression levels of IL18, STAT4, and GPI in peripheral blood of DLBCL patients and healthy controls determined by PCR. **(B)** mRNA expression levels of IL18, STAT4, and GPI in normal B cell lines and DLBCL cell lines detected by PCR. **(C)** Protein expression levels of IL18, STAT4, and GPI in normal B cell lines and DLBCL cell lines detected by Western blot. **(D)** Increased mRNA expression of STAT4 in OCI-LY19 and SU-DHL-2 cells after STAT4 overexpression (OE) compared to control cell lines. **(E)** Elevated protein expression of STAT4 in OCI-LY19 and SU-DHL-2 cells after STAT4 overexpression. **(F)** STAT4 overexpression reduces PCNA (proliferation protein) and N-cadherin (cell migration protein) expression, while increasing E-cadherin (cell adhesion protein) expression in DLBCL cells. *Significant differences were indicated as follows: *P<0.05, **P<0.01, ***P<0.001, ****P<0.0001. ns, not significant.

Following the multivariate analysis, the three genes (IL-18, STAT4, GPIP) exhibiting high P values were chosen for additional experimental validation. The mRNA expression of these genes was then assessed in peripheral blood samples obtained from 41 DLBCL patients and 21 healthy individuals. Comprehensive patient information is provided in **Table S8**. The results revealed a significant down-regulation of IL18 and GPI, accompanied by an up-regulation of STAT4, in peripheral blood samples of DLBCL patients compared to normal samples (**Figure 8A**). In DLBCL cell lines, the mRNA expression levels of IL18, and GPI varied among different cell lines (**Figure 8B**). However, STAT4 consistently exhibited downregulation at both the mRNA and protein levels in DLBCL cell lines, particularly in SU-DHL-2 and OCI-LY19 (**Figures 8B, C**). To investigate the role of STAT4 in DLBCL, we constructed overexpression plasmids to up-regulate STAT4 expression in SU-DHL-2 and OCI-LY19 cells. PCR and western blot analysis confirmed the significant up-regulation of STAT4 expression in the overexpression group (OE-STAT4) compared to the negative control group (Control) and the blank control group (OE-NC) in both cell lines (**Figures 8D, E**). Furthermore, we examined the impact of STAT4 overexpression on cell proliferation and migration by assessing the expression of marker proteins PCNA, E-cadherin, and N-cadherin (**Figure 8F**). Western blot analysis revealed that overexpression of STAT4 significantly reduced the expression of the cell proliferation protein PCNA, increased the expression of the cell adhesion protein E-cadherin, and decreased the expression of the cell migration protein N-cadherin.

### The overexpression of the lactate key gene STAT4 promoted the apoptosis of DLBCL cells

**Figure 9:**
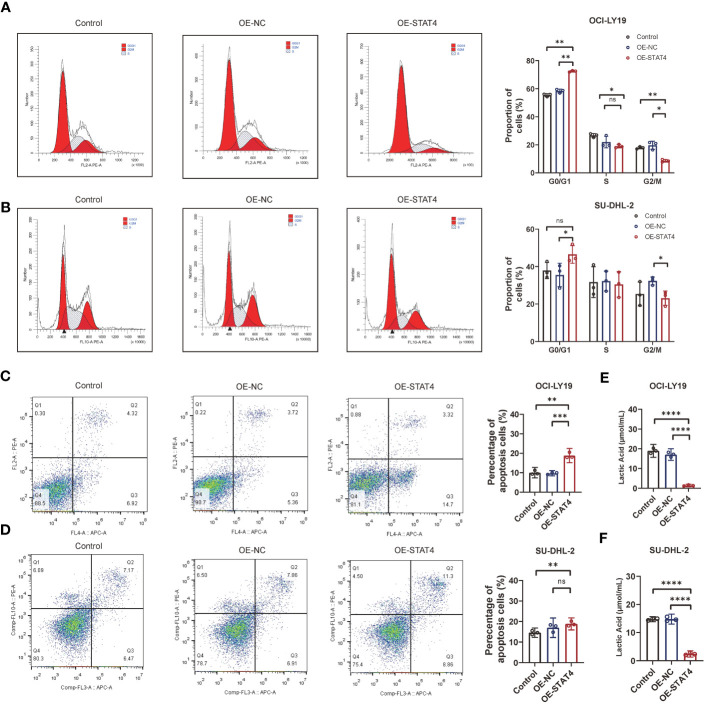
STAT4 overexpression induces cell cycle arrest and enhances cell apoptosis. **(A)** Flow cytometry analysis showing a significant increase in the proportion of cells in the G1 phase and decrease in the proportion of cells in the S and G2/M phase following STAT4 overexpression in OCI-LY19 cells. **(B)** Flow cytometry analysis showing a significant increase in the proportion of cells in the G1 phase and decrease in the proportion of cells in the G2/M phase following STAT4 overexpression in SU-DHL-2C cells. **(C)** STAT4 overexpression significantly promotes apoptosis in OCI-LY19 cells. **(D)** STAT4 overexpression significantly promotes apoptosis in SU-DHL-2C cells. **(E)** Lactate content in OCI-LY19 cells significantly decreases after STAT4 overexpression. **(F)** Lactate content in SU-DHL-2C cells significantly decreases after STAT4 overexpression. *Significant differences were indicated as follows: *P<0.05, **P<0.01, ***P<0.001, ****P<0.0001. ns, not significant.

Additionally, the impact of STAT4 on the cell cycle and apoptosis was assessed through flow cytometry. The results indicated that the upregulation of STAT4 led to a reduction in the percentage of cells in the S phase in OCI-LY19 cells (**Figure 9A**) rather than SU-DHL-2C cells. This discrepancy may be attributed to variations in cell cycle regulation and gene expression patterns in different cancer cell lines. Conversely, there was an increase in the proportion of cells in the G0/G1 phase in both OCI-LY19 and SU-DHL-2C cells (**Figure 9B**). Besides, compared to the other two control groups, STAT4 overexpression promoted more cell apoptosis in both OCI-LY19 cell and SU-DHL-2C cell (**Figures 9C, D**). Finally, the effect of STAT4 expression on lactate content in DLBCL cells was analyzed. The results showed that STAT4 overexpression significantly decreased lactate levels in both OCI-LY19 cell and SU-DHL-2C cells (**Figures 9E, F**), suggesting that STAT4 may play a key role in the relationship between lactate levels in the tumor microenvironment and the development of DLBCL.

### Correlation between STAT4 transcription expression, DNA methylation, and immunomodulators

**Figure 10:**
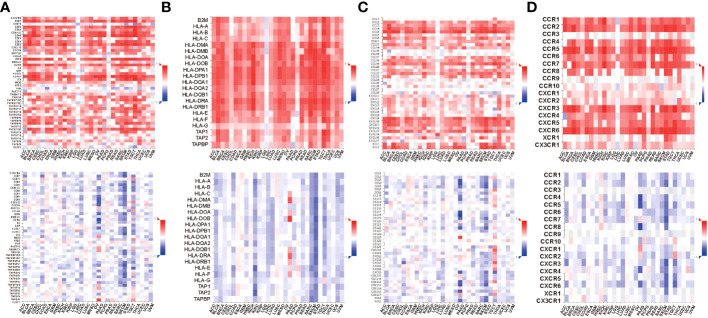
Relations between immunomodulators and expression or methylation of STAT4. **(A)** Spearman correlations between immunomodulators and expression (above), methylation(below) of STAT4. **(B)** Spearman correlations between MHC molecule and expression (above), methylation(below) of STAT4. **(C)** Spearman correlations between Chemokine and expression (above), methylation(below) of STAT4. **(D)** Spearman correlations between Receptor and expression (above), methylation(below) of STAT4.

To obtain additional knowledge regarding the association among STAT4 transcription expression, DNA methylation, and immune markers, we utilized the TISDB online database to assess it. The results revealed that STAT4 transcript expression exhibited a positive correlation with immunomodulators, including C10orf54, CD27, CD28, CD40, CD40LG, CD48, CD70, CD80, CD86, CXCL12, CXCR4, ENTPD1, ICOS, IL2RA, KLRC1, KLRK1, LTA, TMIGD2, TNFRSF13B, TNFRSF17, TNFRSF18, TNFRSF25, TNFRSF4, TNFRSF8, TNFRSF9, and TNFSF13B (**Figure 10A**). Conversely, STAT4 DNA methylation displayed a negative correlation with these immunomodulators. Similarly, STAT4 transcript expression demonstrated a positive correlation with most MHC-related molecules, while STAT4 DNA methylation showed a negative correlation with these molecules (**Figure 10B**). As for the chemokines and receptors, the expression of STAT4 transcripts was positively correlated with CCL2, CCL3, CCL4, CCL5, CCL8, CCL11, CCL13, CCL14, CCL17, CCL18, CCL19, CCL21, CCL22, CXCL9, CXCL10, CXCL11, CXCL12, CXCL13, CXCL16, XCL1, XCL2, CCR1, CCR2, CCR4, CCR5, CCR6, CCR7, CXCR3, CXCR4, CXCR5, and CXCR6, whereas STAT4 DNA methylation exhibited a significantly negative correlation with most chemokines and receptors (**Figures 10C, D**). There is a strong association between immune-related molecules and STAT4 transcription expression in these findings, and STAT4 DNA methylation consistently exhibit opposing trends, suggesting that STAT4 methylation impacts its expression and influences the immune level and degree of cell chemotaxis it regulates.

## Discussion

In this study, we embarked on a comprehensive analysis of histology-specific lactate-related gene expression data from DLBCL samples, and develop a novel prognostic gene signature. Compare with other four published models (13–16), the clinical usefulness of our novel prognostic model was better. Our findings revealed a significant correlation between the prognostic lactate score and various key factors including patient outcomes, immune infiltration levels, and activation of cancer-related pathways in DLBCL. Notably, the prognostic lactate score demonstrated a strong association with the response to R-CHOP treatment and chemotherapeutic drug sensitivity in DLBCL patients, indicating the potential of LAR score as a promising biomarker for guiding treatment in DLBCL.

There are several important findings in this study. First, our study showed that higher lactate scores in DLBCL samples, reflecting increased immunosuppression, corresponded to worse prognosis. Our immune infiltration analysis unveiled insights: low lactate score samples had elevated CD8+ T cells and NK cells. High lactate score subtypes showed the opposite immune cell pattern, hinting at lactate’s potential immunosuppressive role in the tumor microenvironment. This concurs with studies linking lactate to promoting myeloid-derived suppressor cells, curbing anti-tumor immunity (17). Notably, lactate-driven microenvironment acidification hampers T cell activity and cytokine production, exacerbating immune response limitations (18). Our findings, coupled with existing literature, collectively support a link between high lactate accumulation and an immunosuppressive cancer microenvironment.

Second, our findings indicate a positive correlation between the lactate score and the MYC signaling pathway, which holds significant implications for tumor immune evasion and immune checkpoint regulation. This pathway can stimulate the expression of immune checkpoint genes, such as CD47 and PDL1, which facilitate tumor immune evasion (19). As the efficacy of ICIs are contingent on the presence of CD8+T cells within tumors (20), the combination of ICIs with therapies that augment the number of CD8+T cells is optimal. Thus, by mitigating lactate-driven immunosuppression while concurrently leveraging ICIs to bolster the immune response, we propose that the combination of lactate inhibitors with ICIs holds immense potential.

In addition, our investigation established the lactate score as a reliable predictor of response to first-line treatment with R-CHOP regimen in DLBCL patients. Previous studies have highlighted the challenges of achieving a cure in 40% to 50% of DLBCL patients following R-CHOP treatment (21). However, our study revealed that patients in the low lactate score group exhibited response rates of up to 82% when treated with R-CHOP regimen. This finding provides valuable insights for treatment selection in DLBCL patients. Additionally, we identified four pairs of drugs, including Entospletinib, IAO 5620, Staurosporine, and Dasatinib, whose drug sensitivity positively correlated with the lactate-related gene signature, indicating increased resistance in the high-risk group. Notably, drugs targeting the SRC and RTK signaling pathways that associated with the increase of lactate content (10, 22), exhibited increased sensitivity in the high-risk group. Drugs targeting the IGF1R and apoptosis pathways that associated with the reduction of lactate content and antitumor effect (23, 24) were negatively correlated with the lactate score, suggesting a potential link between lactate-related molecules and chemoresistance mechanisms in DLBCL. These findings shed light on the role of lactate metabolism in influencing the effectiveness of chemotherapy and provide insights into novel strategies for overcoming chemoresistance in DLBCL.

Finally, our findings highlight the potential of targeting lactate-related genes, particularly STAT4, as a promising therapeutic strategy in DLBCL. Previous study demonstrated that STAT4 plays an important role in antitumor immunity (25). Through STAT4-mediated signal transduction, IL-12 could produce an autocrine and paracrine antigen that relies on interferon-gamma (IFN-g) to enhance antitumor immunity (26). This molecular mechanism can promote the immune system of the body to fight tumors, while its anticancer effect in DLBCL cell has not been reported. In this study, we first observed elevated mRNA and protein expression of STAT4 in DLBCL cell lines. Subsequently, we showed that STAT4 overexpression could significantly inhibit proliferation and migration, promote apoptosis, and reduce the lactate content of DLBCL cells, suggesting that targeting STAT4 may effectively reduce lactate in the tumor microenvironment and exert an antitumor therapeutic effect. It should be noted that the expression of STAT4 mRNA was reduced in peripheral blood samples of DLBCL patients, this discrepancy may be attributed to the regulatory influence exerted by peripheral blood’s diverse cell types on STAT4 expression. Furthermore, the correlation between STAT4 transcript expression and DNA methylation with a multitude of immunomodulators, as well as numerous chemokines and receptors listed in TISIDB, offers valuable insight into the intricate immune landscape of DLBCL. Although our study has provided valuable insights into the association between transcriptional expression and methylation patterns, further research is required to elucidate the underlying mechanisms driving this correlation.

We recognize some limitations of our research. Although we have largely corrected for batch effects, the heterogeneity of tumor samples biased the expression of lactic-related genes to some extent. We are in the process of collecting samples from a multicenter clinical cohort for further analysis and validation. Additionally, although we have performed some cell validation experiments, further *in vitro* and *in vivo* experiments are needed to determine the molecular mechanism of lactate molecules in antitumor immunity.

## Conclusion

Lactate-related gene signature enables evaluation of DLBCL clinical significance, immune infiltration, and therapeutic benefit. The involvement of key lactate gene STAT4 in DLBCL cells leads to the inhibition of proliferation and migration, induction of cell cycle arrest, and promotion of cell apoptosis. Modulating STAT4 could be a promising strategy for DLBCL in clinical practice.

## Data availability statement

The original codes used for analyses presented in the study are publicly available. This data can be found here: https://github.com/gdphG/R-code.git.

## Ethics statement

The studies involving humans were approved by the ethics review committee of Guangdong Provincial People’s Hospital. The studies were conducted in accordance with the local legislation and institutional requirements. The participants provided their written informed consent to participate in this study. Ethical approval was not required for the studies on animals in accordance with the local legislation and institutional requirements because only commercially available established cell lines were used.

## Author contributions

JW and YW acquired the data, performed the analysis, and wrote the manuscript. LW participated in data analysis. XC and HZ were responsible for data curation. LZ and SY were involved in study design, supervision, and acquiring funding. All authors contributed to the study conception and designed and read and approved the final manuscript.

## Supplementary material

Supplementary Figure 1 Prognostic differences between wild-type and mutant types of lactate gene.

Supplementary Figure 2 The DCA curve for the overall survival rate at 1 year, 2 years, 3 years, and 5 years for the risk model 1 and other risk model. Model 1: LAR score model; Model 2: metabolism-related risk model; Model 3: immune-related risk model; Model 4: prognostic-related risk model; Model 5: Cachexia-inducing factors (CIFs)-related risk model.

Supplementary Figure 3 Correlation between LARrisk and TIDE. **(A)** Analysis of differences in TIDE values between high and low-risk groups in the GSE181063 dataset. **(B)** Difference analysis of TIDE values between high and low-risk groups in the GSE32918 dataset. **(C)** Difference analysis of TIDE values between high and low-risk groups in the GSE10846 dataset. **(D)** Analysis of differences in TIDE values between high and low-risk groups in the GSE69053 dataset. **(E)** Correlation test between risk score and TIDE value in the high and low-risk groups across the four datasets.
