# Supplementary figures and images for: Identification of lactate regulation pattern on tumor immune infiltration, therapy response, and DNA methylation in diffuse large B-cell lymphoma
Source: Front Immunol. 2023 Sep 18;14:1230017. doi: 10.3389/fimmu.2023.1230017 (PMC10542897; doi:10.3389/fimmu.2023.1230017)

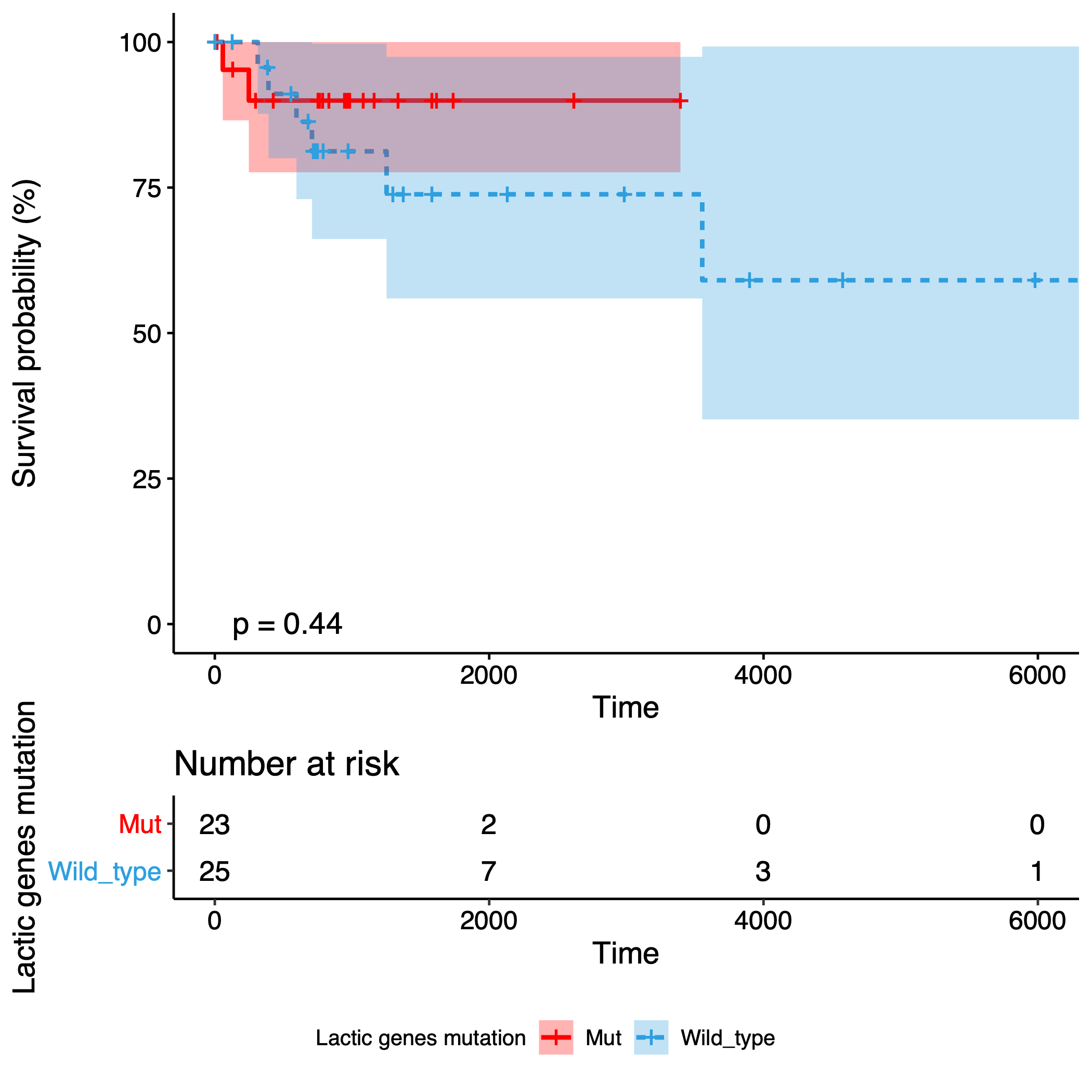

Supplement: Supplementary Figure 1 — Prognostic differences between wild-type and mutant types of lactate gene. [file Image_1.tiff]

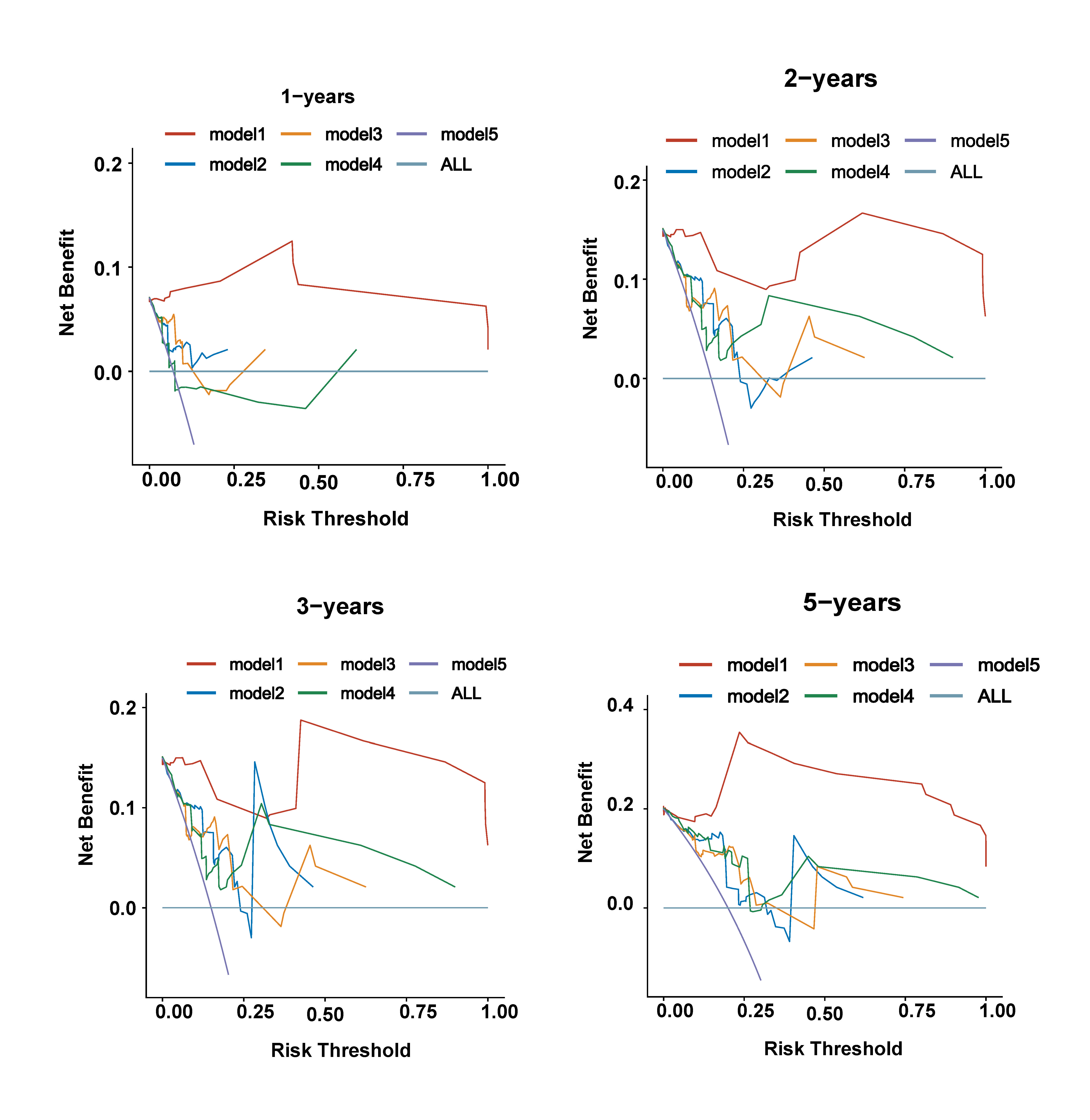

Supplement: Supplementary Figure 2 — The DCA curve for the overall survival rate at 1 year, 2 years, 3 years, and 5 years for the risk model 1 and other risk model. Model 1: LAR score model; Model 2: metabolism-related risk model; Model 3: immune-related risk model; Model 4: prognostic-related risk model; Model 5: Cachexia-inducing factors (CIFs)-related risk model. [file Image_2.tif]

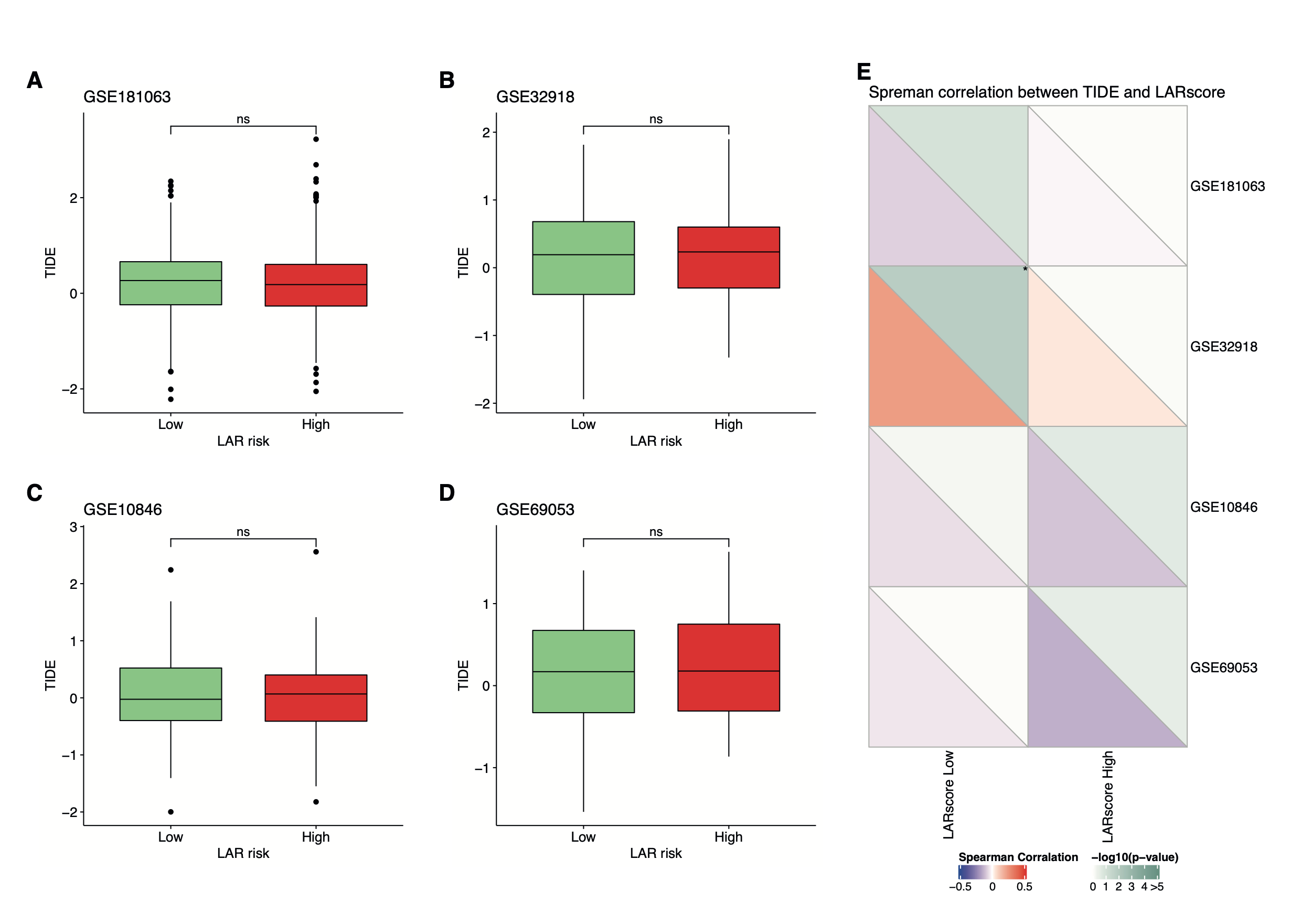

Supplement: Supplementary Figure 3 — Correlation between LARrisk and TIDE. (A) Analysis of differences in TIDE values between high and low-risk groups in the GSE181063 dataset. (B) Difference analysis of TIDE values between high and low-risk groups in the GSE32918 dataset. (C) Difference analysis of TIDE values between high and low-risk groups in the GSE10846 dataset. (D) Analysis of differences in TIDE values between high and low-risk groups in the GSE69053 dataset. (E) Correlation test between risk score and TIDE value in the high and low-risk groups across the four datasets. [file Image_3.tif]
